# Association of Psychological Resilience and Depressive Symptoms in Adults With Cardiac Disease

**DOI:** 10.1093/geroni/igaa057.2819

**Published:** 2020-12-16

**Authors:** Amy Ketcham, Austin Matus, Barbara Riegel

**Affiliations:** University of Pennsylvania, Philadelphia, Pennsylvania, United States

## Abstract

Depressive symptoms predict hospitalization and mortality in adults with cardiac disease. Psychological resilience may protect against depressive symptoms, but benefits are not yet conclusive. We conducted a systematic review to examine the association between resilience and depressive symptoms in adults with cardiac disease. Seven databases were searched from inception to December 2019 using the search terms “cardiac disease,” “depressive symptoms,” “depression,” and “resilience.” The 623 articles identified were narrowed through title, abstract and full-text review leaving 13 studies for final analysis. Resilience and depressive symptoms were inversely related in 10 of 13 studies. The three studies with poor quality sampling techniques or significant loss to follow-up found null results. The major gap identified in the literature was poor understanding of the longitudinal pattern between resilience and depressive symptoms. If the direction of causality functions as expected in longitudinal research, optimizing resilience could help attenuate depressive symptoms.

